# Farnesoid X Receptor and Liver X Receptor Ligands Initiate Formation of Coated Platelets

**DOI:** 10.1161/ATVBAHA.117.309135

**Published:** 2017-07-26

**Authors:** Amanda J. Unsworth, Alexander P. Bye, Dionne S. Tannetta, Michael J.R. Desborough, Neline Kriek, Tanya Sage, Harriet E. Allan, Marilena Crescente, Parveen Yaqoob, Timothy D. Warner, Chris I. Jones, Jonathan M. Gibbins

**Affiliations:** From the Institute of Cardiovascular and Metabolic Research, School of Biological Sciences (A.J.U., A.P.B., N.K., T.S., M.C., C.I.J., J.M.G.) and Department of Food and Nutritional Sciences (D.S.T., P.Y.), University of Reading, United Kingdom; Oxford Haemophilia and Thrombosis Centre, Oxford Biomedical Research Centre, Churchill Hospital, United Kingdom (M.J.R.D.); Nuffield Division of Clinical Laboratory Sciences, University of Oxford, United Kingdom (M.J.R.D.); and Blizard Institute, Barts & the London School of Medicine & Dentistry, United Kingdom (H.E.A., M.C., T.D.W.).

**Keywords:** bile, blood coagulation, blood platelets, calcium, cholesterol

## Abstract

**Objectives—:**

The liver X receptors (LXRs) and farnesoid X receptor (FXR) have been identified in human platelets. Ligands of these receptors have been shown to have nongenomic inhibitory effects on platelet activation by platelet agonists. This, however, seems contradictory with the platelet hyper-reactivity that is associated with several pathological conditions that are associated with increased circulating levels of molecules that are LXR and FXR ligands, such as hyperlipidemia, type 2 diabetes mellitus, and obesity.

**Approach and Results—:**

We, therefore, investigated whether ligands for the LXR and FXR receptors were capable of priming platelets to the activated state without stimulation by platelet agonists. Treatment of platelets with ligands for LXR and FXR converted platelets to the procoagulant state, with increases in phosphatidylserine exposure, platelet swelling, reduced membrane integrity, depolarization of the mitochondrial membrane, and microparticle release observed. Additionally, platelets also displayed features associated with coated platelets such as P-selectin exposure, fibrinogen binding, fibrin generation that is supported by increased serine protease activity, and inhibition of integrin αIIbβ3. LXR and FXR ligand-induced formation of coated platelets was found to be dependent on both reactive oxygen species and intracellular calcium mobilization, and for FXR ligands, this process was found to be dependent on cyclophilin D.

**Conclusions—:**

We conclude that treatment with LXR and FXR ligands initiates coated platelet formation, which is thought to support coagulation but results in desensitization to platelet stimuli through inhibition of αIIbβ3 consistent with their ability to inhibit platelet function and stable thrombus formation in vivo.

## Abstract 2

Supplemental Digital Content is available in the text.

Platelets act as the first line of defense after vascular injury and play a key role in the prevention of excessive blood loss through the formation of a thrombus at the site of vessel damage. Unwanted, excessive platelet activation and hyper-reactive platelets are thought to contribute to atherothrombosis that can lead to myocardial infarction and stroke. Several pathological conditions such as hyperlipidemia,^1,2^ type 2 diabetes mellitus,^3,4^ metabolic syndrome,^5^ obesity,^5,6^ and high cholesterol^7–12^ have been related to platelet hyper-reactivity and increased platelet responses.

Formation of procoagulant platelets is one way in which platelets can be classed as hyper-reactive. A subclass of procoagulant platelets are coated platelets (previously called COAT platelets)^13^ that expose and retain high levels of phosphatidylserine and high levels of α-granule proteins on their surface including factor V, fibrinogen, fibronectin, and von Willebrand factor.^14–16^ Coated platelets are also associated with deregulated levels of intracellular calcium, depolarized mitochondrial membranes, generation of reactive oxygen species (ROS), loss of membrane integrity, and the release of platelet microparticles.^17–19^ Coated platelets are reported to support coagulation reactions, whereas cleavage and inactivation of proteins required for integrin signaling prevents aggregation and inhibits traditional platelet activation in response to platelet agonists.^20,21^

Several intracellular nuclear receptors that have been identified in human platelets such as the liver X receptor (LXR) and farnesoid X receptor (FXR), are recognized for their roles in the transcriptional regulation of metabolic pathways that are disrupted in conditions such as type II diabetes mellitus, metabolic syndrome, and hyperlipidemia.^22–27^ The LXR receptors are involved in the regulation of cholesterol homeostasis and their natural ligands, for example, oxysterols, are cholesterol derivatives.^28–33^ Cholesterol derivatives have been found to deregulate platelet responses to several platelet agonists, with some derivatives potentiating and others inhibiting aggregation.^34–36^ Similarly, the FXR receptor is activated by bile acids and other cholesterol derivatives with roles in the regulation of bile acid and cholesterol homeostasis^37–39^ and has also been shown to play a role in insulin homeostasis.^40,41^ Bile acids have previously been associated with platelet dysfunction^42^ and treatment of platelets with bile acids has been shown to be associated with membrane vesiculation and platelet swelling.^43,44^

Increasing evidence supports nongenomic actions for intracellular nuclear receptors, including FXR and LXR, that are clearly distinct from their well-established genomic roles.^27,45,46^ Previous work has shown that ligands for both LXR and FXR inhibit platelet activation by GPVI (glycoprotein VI) agonists and thrombin, as evidenced by inhibition of platelet aggregation, granule secretion, and calcium mobilization in response to collagen receptor GPVI agonists and thrombin. Furthermore outside-in signaling through integrin αIIbβ3 is inhibited resulting in a significant reduction in thrombus formation and stability in vivo,^25,27^ although the mechanisms by which these ligands cause this inhibition seem to be different.^25,27^

The ability of LXR and FXR ligands to inhibit platelet function seems at odds with the observations that conditions associated with increased circulating levels of LXR or FXR ligands, such as hyperlipidemia, type 2 diabetes mellitus, metabolic syndrome, obesity, and high cholesterol are associated with increased platelet reactivity and an increased risk of cardiovascular disease.^1–12^ Furthermore, many natural and synthetic LXR and FXR ligands including hydroxycholesterols and bile acids have been shown to be cytotoxic and proapopotic in some cell types.^47–53^ We have also previously observed that platelets treated with high concentrations of FXR ligand GW4064 showed decreased sample turbidity indicative of platelet swelling and procoagulant activity.^27^ Therefore, despite previous observations that treatment of platelets with LXR or FXR ligands inhibits platelet function to platelet agonists, understanding how these nuclear receptor ligands nongenomically affect platelets before agonist stimulation is of clear importance.

It has been previously described that during thrombus formation 2 distinct populations of platelets appear, coaggregated platelets, which support thrombus growth, and loosely attached platelets that expose phosphatidylserine, which support coagulation but cannot support platelet activation.^20,54^ We set out to determine whether LXR and FXR ligands can both activate and inhibit platelets.

In this study, we sought to explore in more detail how LXR and FXR ligands may affect the ability of platelets to respond to platelet agonists. We identify that treatment of platelets with LXR or FXR ligands can convert platelets to a procoagulant state that is consistent with coated platelet formation.

## Materials and Methods

Materials and Methods are available in the online-only Data Supplement.

## Results

### LXR and FXR Ligands Increase Procoagulant Activity of Platelets

**Figure 1.**
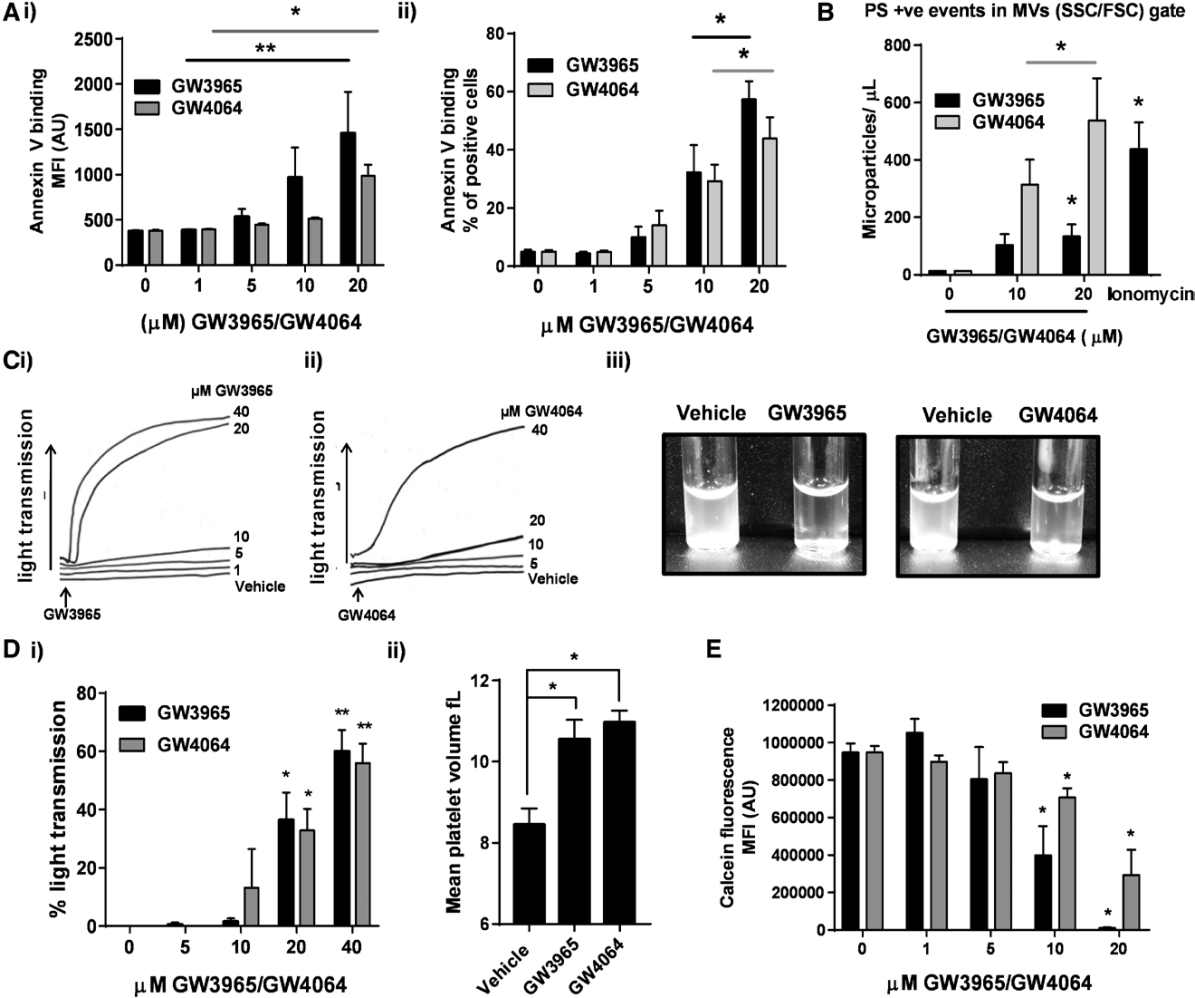
Liver X receptor (LXR) and farnesoid X receptor (FXR) ligands induce platelet procoagulant activity. Human washed platelets were treated for 10 min with or without increasing concentrations of GW3965 (1, 5, 10, 20, and 40 µmol/L) or GW4064 (1, 5, 10, 20, and 40 µmol/L) or vehicle control before analysis by flow cytometry for (**A**) annexin V binding, which is a measure of phosphatidylserine exposure, and data expressed as (i) median fluorescence intensity and (ii) percentage of annexin V–positive cells; (**B**) formation of microparticles, determined by gating for the microvesicle population using forward and side scatter profiles of ApogeeMix beads. Data expressed as number of phosphatidylserine (PS)–positive events per microliter. **C** and **D**, Platelet swelling, measured by analyzing (**C**) light transmission, with an increase in transmission associated with increased platelet swelling (i) and (ii) representative traces and (iii) representative images. **D**, (i) data expressed as % of light transmission; (ii) mean platelet volume. **E**, Calcein fluorescence, to measure membrane integrity, expressed as median fluorescence intensity. Results are mean+SEM for n≥3. *P<0.05 in comparison to vehicle controls.

We set out to determine whether treatment with the LXR or FXR ligands (using concentrations previously shown to inhibit platelet function) was able to convert resting platelets to the procoagulant state. To do this, several markers of procoagulant activity were measured, including phosphatidylserine exposure, microparticle formation, platelet swelling, and loss of membrane integrity. Phosphatidylserine exposure was determined by measuring annexin V binding after treatment of washed platelets with either the LXR ligand GW3965 or FXR ligand GW4064 (1, 5, 10, and 20 µmol/L) for 10 minutes. Platelets treated with either GW3965 or GW4064 showed a concentration-dependent increase in phosphatidylserine exposure with increases in the median fluorescence intensity (Figure 1Ai) and percentage of positive cells (Figure 1Aii) for annexin V binding observed (gating strategy shown in Figure IA in the online-only Data Supplement). Microparticle formation from washed platelets (Figure 1B) was also increased in a concentration-dependent manner in GW3965- or GW4064-treated platelets in comparison to vehicle-treated controls (gating strategy shown in Figure IB through ID in the online-only Data Supplement). Platelet swelling (Figure 1C and 1D), measured as a decrease in sample turbidity and an increase of mean platelet volume (using an ACT5 hematology analyzer) was also evident after treatment with either the LXR or FXR ligands (5, 10, 20, and 40 µmol/L). The observed decrease in sample turbidity was not because of aggregation as it could not be prevented by pretreatment with integrilin (5 µmol/L, a concentration shown to block fibrinogen binding and aggregation), and cell lysis was excluded as treatment with GW3965 or GW4064 did not reduce platelet count (Figure II in the online-only Data Supplement).^20^ Finally, membrane integrity (Figure 1E) determined by measuring calcein permeability was also altered after treatment with either GW3965 or GW4064. Both compounds caused a decrease in calcein fluorescence, indicating that the integrity of the platelet membrane had been compromised, as has been previously observed during procoagulant platelet formation.^21,55,56^ In support of these observations being the result of specific activity of the LXR and FXR receptors, concentration-dependent increases in annexin V binding were also observed after treatment with natural ligands for the LXR receptor 27-hydroxycholesterol and 24S-hydroxycholesterol (10, 30, and 100 µmol/L) or natural ligand for the FXR receptor chenodeoxycholic acid (100 and 300 µmol/L; Figure III in the online-only Data Supplement).

### LXR and FXR Ligands Initiate Formation of Coated Platelets

**Figure 2.**
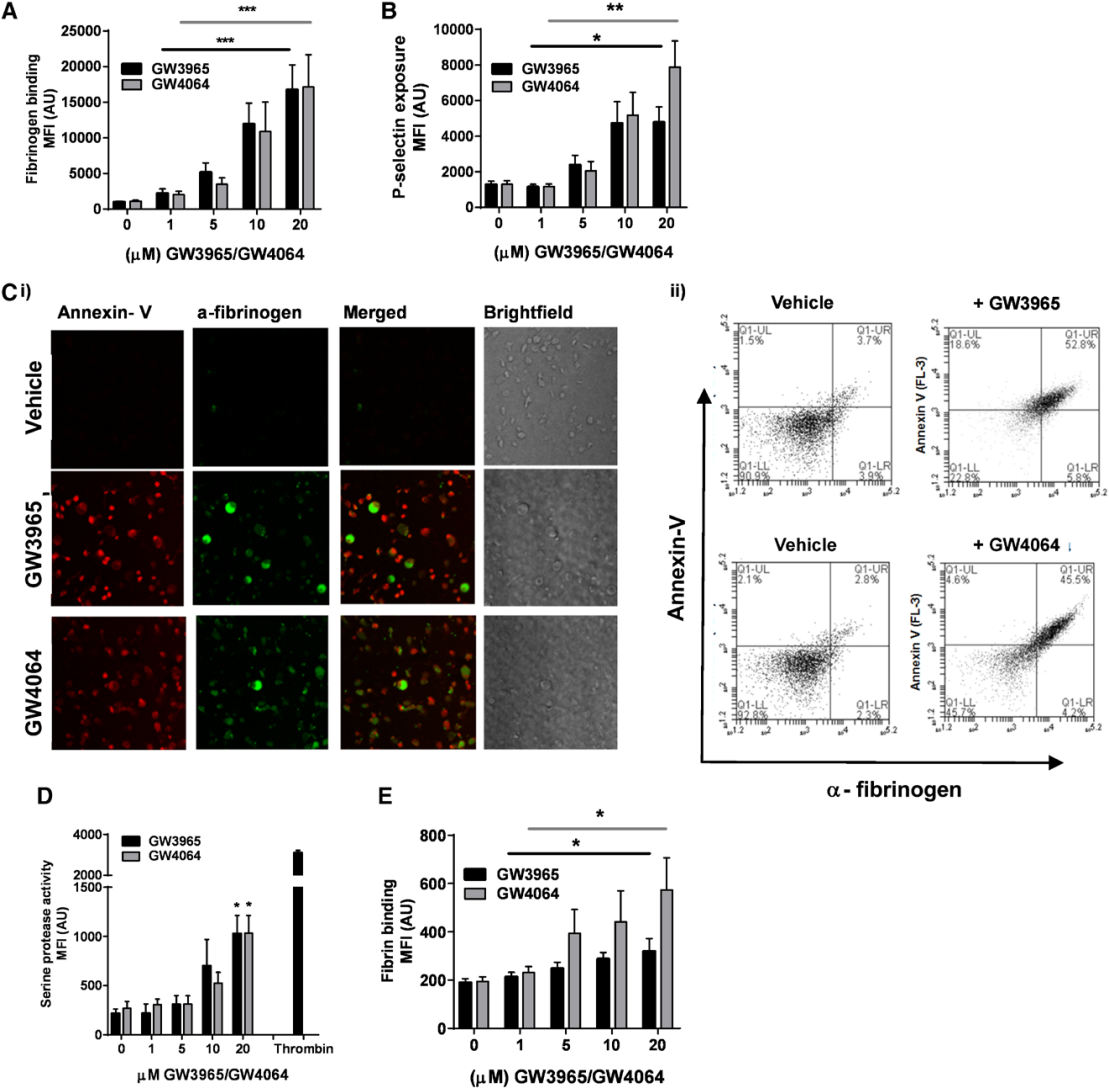
Liver X receptor (LXR) and farnesoid X receptor (FXR) agonists initiate coated platelet formation. Human washed platelets were treated with increasing concentrations of GW3965 (1, 5, 10, and 20 µmol/L), GW4064 (1, 5, 10, and 20 µmol/L), or vehicle control and tested for several different markers of coated platelet formation including (**A**) fibrinogen binding, (**B**) α-granule components, measured by detecting P-selectin exposure, (**D**) serine protease activity, measured by quantification of release of fluorescence as a result of conversion of Z-Gly-Gly-Arg-AMC fluorescent substrate for 1 h at 37°C, and (**E**) bound fibrin. Data expressed as median fluorescence intensity. **C**, Human platelets treated with GW3965 (20 µmol/L), GW4064 (20 µmol/L), or vehicle were stained with annexin V–Cy5 (in red) and antifibrinogen (Alexa 488 conjugated; in green) and analyzed by (i) fluorescence microscopy on a ×100 oil immersion lens and (ii) flow cytometry. (i) Representative images and (ii) representative scatter plots shown. Other results are mean+SEM for n≥3. *P<0.05 in comparison to vehicle controls.

Coated platelets are procoagulant platelets that in addition to exposure of phosphatidylserine are characterized by high levels of surface retention and exposure of α-granule proteins including factor V, fibrinogen, fibronectin, and von Willebrand factor. To determine whether GW3965 and GW4064 were capable of stimulating coated platelet formation, fibrinogen binding, and P-selectin exposure were measured after treatment with either ligand. Washed platelets treated with either the LXR or FXR ligand (1, 5, 10, and 20 µmol/L) exhibited a concentration-dependent increase in fibrinogen binding and P-selectin exposure in comparison to vehicle-treated controls (Figure 2A and 2B). Because no exogenous fibrinogen was added to the assay, we hypothesize that the fibrinogen bound at the platelet surface comes from the activated platelet via α-granule secretion, which also leads to increased P-selectin exposure at the platelet surface. As with annexin V binding, treatment of platelets with the natural ligands for the LXR and FXR receptors, 27-hydroxycholesterol and 24S-hydroxycholesterol (10, 30, and 100 µmol/L) or chenodeoxycholic acid (100 and 300 µmol/L) also caused an increase in fibrinogen binding, further supporting that these observations are because of specific activity of the LXR and FXR receptors (Figure III in the online-only Data Supplement).

In further support of procoagulant coated platelet formation, immunofluorescence microscopy of platelets stained with annexin V and an antifibrinogen antibody confirmed the formation of phosphatidylserine exposing ballooned platelets that bind fibrinogen after treatment with GW3965 (20 µmol/L) and GW4064 (20 µmol/L) in comparison to vehicle-treated controls (Figure 2Ci). In further support of this, imaging flow cytometry confirmed that GW3965- and GW4064-treated platelets show an increase in fibrinogen and annexin V binding compared with vehicle-treated control (Figure IV in the online-only Data Supplement), and single cell flow cytometry 2 feature dot plot analysis showed that platelets found to be positive for annexin V binding were also found to bind fibrinogen. This is shown in Figure 2Cii, with an increased number of events observed in the upper right quadrant of the dot plot after treatment with GW3965 or GW4064 compared with vehicle control (Figure 2Cii).

Increases in platelet prothrombinase activity and the subsequent conversion of fibrinogen into fibrin is another key feature of coated procoagulant platelets.^57^ The ability of the LXR and FXR agonists to induce platelet prothrombinase activity was quantified as the rate of cleavage of Z-Gly-Gly-Arg-AMC, a serine protease substrate. Both GW3965- and GW4064-treated platelets showed a significant increase in serine protease activity in comparison to vehicle controls (Figure 2D). Although this is likely to reflect assembly of the prothrombinase complex at the cell surface, the action of other coagulation enzymes may also contribute. In support of this increase in serine protease activity being because of increased thrombin activity, a dose-dependent increase in surface fibrin, detected using an antifibrin antibody, was also observed after treatment with either GW3965 or GW4064 (Figure 2E). Additionally, analysis of thrombin generation in platelet-rich plasma by calibrated automated thrombography found that treatment with either GW3965 or GW4064 reduced the time taken to reach peak thrombin generation compared with vehicle-treated controls (Figure V in the online-only Data Supplement). Together, these observations support a role for both LXR and FXR ligands in the regulation of platelet prothrombinase (serine protease) activity and fibrin generation.

### LXR and FXR Ligand-Dependent Formation of Procoagulant Platelets Inhibits Activation of Integrin αIIbβ3

**Figure 3.**
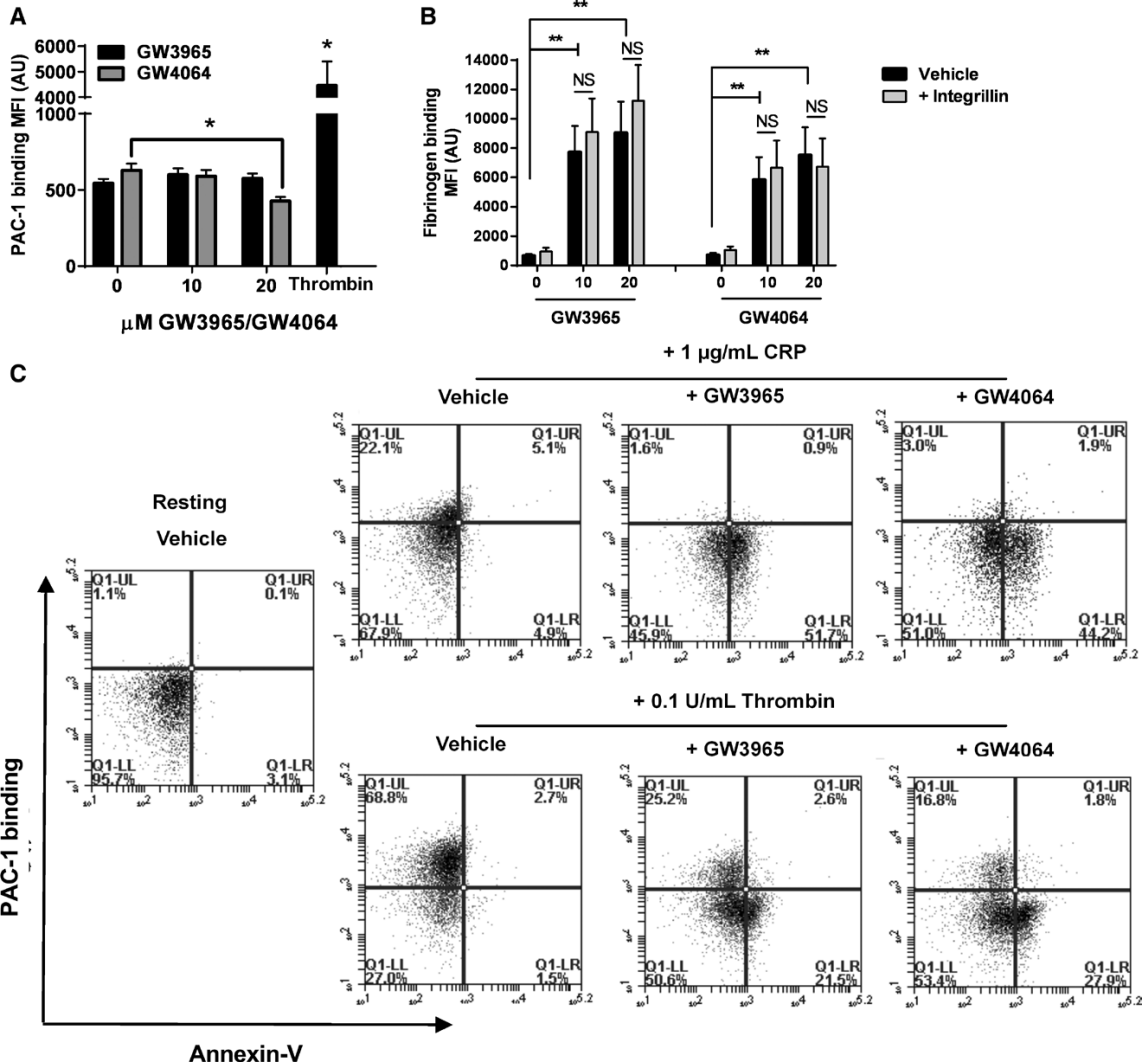
Liver X receptor (LXR) and farnesoid X receptor (FXR) agonists inhibit αIIbβ3. Human washed platelets were prepared, and the effect of GW3965 or GW4064 (10 and 20 µmol/L) on (**A**) integrin αIIbβ3 activation determined by PAC-1 antibody binding and (**B**) fibrinogen binding in the presence or absence of integrillin (5 µmol/L), an inhibitor of integrin αIIbβ3 fibrinogen binding. (Data expressed as median fluorescence intensity, mean+SEM for n≥3. **C**, PAC-1 binding and phosphatidylserine exposure in CRP-stimulated (1 µg/mL) and thrombin-stimulated (0.1 U/mL) platelets was determined. Representative scatter plots shown.

It has been shown that fibrinogen binding to coated platelets is independent of activation of integrin αIIbβ3.^16,20^ We, therefore, tested whether the observed increase in fibrinogen binding in GW3965- or GW4064-treated platelets was associated with activation of integrin αIIbβ3. In support of integrin αIIbβ3 not being activated, neither GW3965 nor GW4064 caused an increase in PAC-1 antibody binding to the open (active) conformation of integrin αIIbβ3 (Figure 3A). Furthermore, pretreatment of platelets with integrilin at a concentration (5 µmol/L) known to block fibrinogen binding to integrin αIIbβ3^27^ was unable to reverse the observed increase in fibrinogen binding (Figure 3B). These results suggest that increased binding of fibrinogen to the platelet surface is independent of integrin αIIbβ3 activation as has been previously described for coated platelet formation.^16,20^

Phosphatidylserine exposure in coated platelets is associated with closure and inhibition of the αIIbβ3 integrin and other platelet receptors.^20^ Interestingly, previously published data have shown that treatment with similar concentrations of GW3965 and GW4064 causes inhibition of platelet aggregation to GPVI agonists and thrombin.^25,27^ In further support of these findings, we observed inhibition of integrin αIIbβ3 function, determined by measuring PAC-1 binding, after treatment with GW3965 and GW4064 (10 and 20 µmol/L) and stimulation with collagen-related peptide (CRP-XL; 1 µg/mL) or thrombin (0.1 U/mL; Figure VIA in the online-only Data Supplement). Treatment with GW3965 and GW4064 also resulted in reduced cell adhesion and spreading on fibrinogen-coated coverslips (Figure VIB and VIC in the online-only Data Supplement). In support of the formation of coated platelets after treatment with GW3965 and GW4064 leading to inhibition of integrin αIIbβ3, dot plot analysis of CRP-stimulated (1 µg/mL) and thrombin-stimulated (0.1 U/mL) platelets shows an increase in phosphatidylserine exposure and a reduction in PAC-1 binding after treatment with GW3965 or GW4064 compared with vehicle controls. In particular, platelets identified as positive for annexin V binding were found to be negative for PAC-1 binding, as shown by lack of events in the upper right quadrant of the dot plot (Figure 3C). Analysis of nonadhered platelets after exposure to fibrinogen-coated coverslips for 45 minutes also showed that GW3965- and GW4064-treated platelets displayed significantly increased levels of annexin V binding in the nonadhered platelets (which is associated with inhibition of adhesion and spreading) compared with vehicle control (Figure VID in the online-only Data Supplement). Finally, we tested whether the observed increase in fibrinogen binding in GW3965- or GW4064-treated platelets was associated with Talin cleavage, a calcium-dependent step in the activation of integrin αIIbβ3. As shown in Figure VIE in the online-only Data Supplement, platelets treated with GW3965 (20 µmol/L) or GW4064 (20 µmol/L) did not show cleavage of the Talin head domain compared with an ionomycin-treated (5 µmol/L) positive control, further suggesting that integrin αIIbβ3 is not activated, leaving αIIbβ3 unable to initiate outside-in signaling.

### GW3965- and GW4064-induced platelet procoagulant activity Is associated With depolarization of the mitochondrial membrane and Is dependent on sustained increases in intracellular calcium

**Figure 4.**
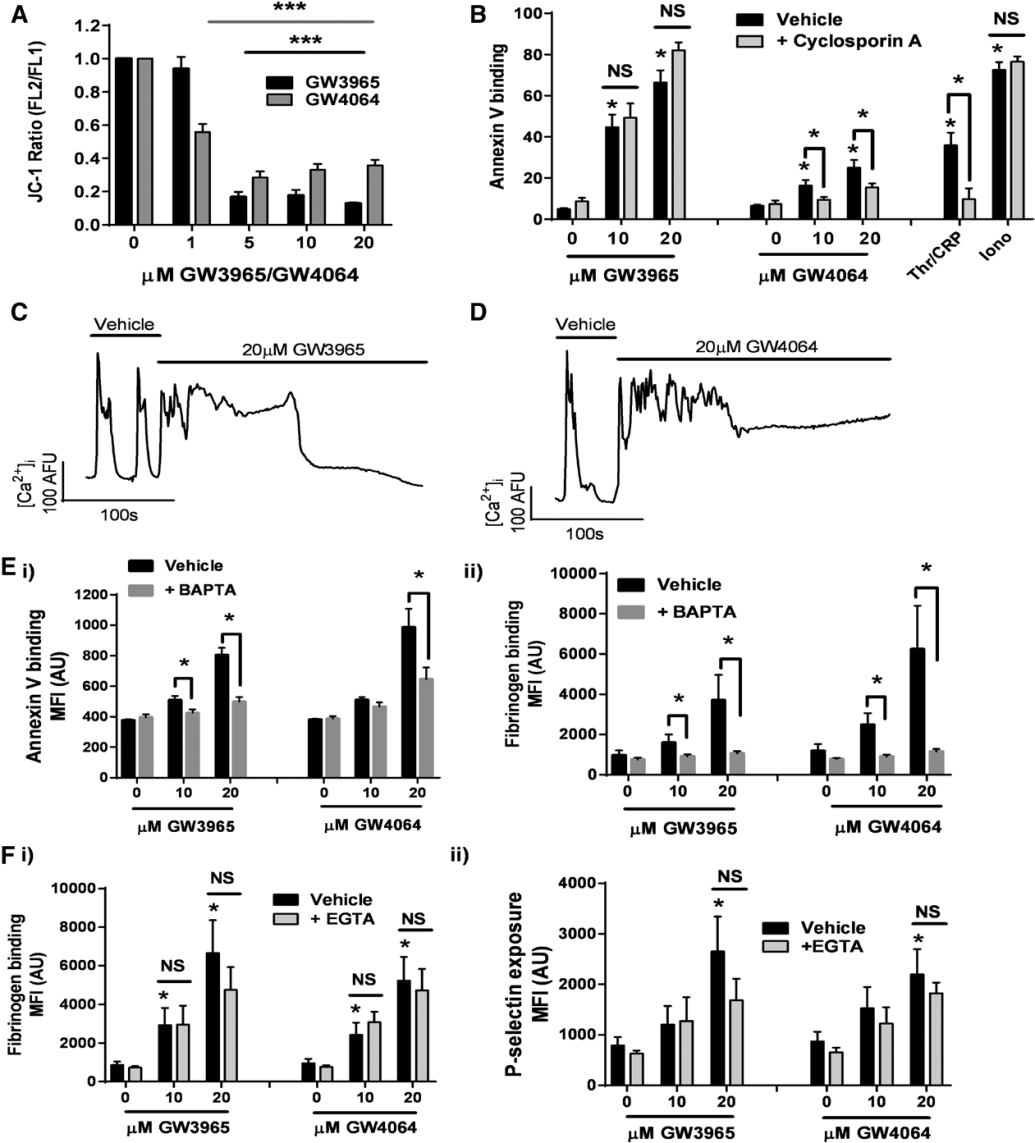
Mitochondrial transmembrane potential and sustained calcium signaling are required for the induction of LXR- and FXR-mediated procoagulant activity. Human washed platelets were treated for 10 min with or without increasing concentrations of GW3965 (1, 5, 10, and 20 µmol/L) or GW4064 (1, 5, 10, and 20 µmol/L) or vehicle control before analysis by flow cytometry for (**A**) changes in the mitochondrial membrane potential, determined by using JC-1 dye and expressing the results using the FL2/FL1 ratio. An increase in the ratio compared with control indicates hyperpolarization of the mitochondrial membrane, whereas a decrease in the FL2/FL1 ratio indicates membrane depolarization. **B**, Platelets were treated for 10 min with or without increasing concentrations of GW3965 (10 and 20 µmol/L) or GW4064 (10 and 20 µmol/L) or vehicle control in the presence of cyclosporine A (5 µmol/L), a cyclophilin D inhibitor, before analysis by flow cytometry for annexin V binding. Data expressed as percentage of annexin V–positive events. Thrombin-treated (0.1 U/mL)/CRP-treated (1 µg/mL) and ionomycin-treated (5 µmol/L) samples were included as positive controls. **C** and **D**, Ca^2+^ imaging in single platelets under flow. Human washed platelets loaded with Fluo4-AM (2 µmol/L) were attached onto mouse antihuman PECAM-1 antibody–coated (WM59) glass-bottom Vena8 GCS biochips (Cellix Ltd, Dublin, Ireland) and vehicle. **C**, GW3965 (20 µmol/L) or (**D**) GW4064 (20 µmol/L) were then flowed through the chips at a slow shear rate of 400 s^−1^ and calcium signaling monitored by observing fluorescence for 5 min. Single platelet, representative calcium signaling trace shown. **E** and **F**, Platelets were treated with GW3965 or GW4064 (0, 10, and 20 µmol/L) in the presence or absence of (**E**) BAPTA (10 µmol/L) or (**F**) EGTA (1 mmol/L) before analysis by flow cytometry for (**E**) annexin V binding, and fibrinogen binding and (**F**) fibrinogen binding and P-selectin exposure. Thr (0.1 U/mL) and Thr (0.1 U/mL)+CRP (5 µg/mL) included as positive controls for fibrinogen binding in the presence of EGTA. Data expressed as median fluorescence intensity. Results are mean+SEM for n≥3 and expressed as percentage of vehicle control. *P<0.05 in comparison to vehicle controls.

Phospholipid scrambling and conversion to the procoagulant state in platelets is attributed to both loss of mitochondrial transmembrane potential and an increase in intracellular calcium.^19,55^ To test whether this was the underlying mechanism behind the observed characteristics of procoagulant platelets induced by the LXR and FXR ligands, the integrity of the mitochondrial membrane was investigated using the JC-1 fluorophore. As shown in Figure 4A in comparison to vehicle control, both GW3965 and GW4064 cause a significant reduction in the JC-1 FL2/FL1 ratio, indicating mitochondrial membrane depolarization. It has also been previously described that cyclophilin D can play a role in formation of procoagulant platelets after stimulation by platelet agonists.^19^ Treatment of platelets with cyclosporine A, a cyclophilin D inhibitor, prevented FXR ligand GW4064-mediated increases in annexin V binding similar to that observed in thrombin- and CRP-stimulated platelets, but phosphatidylserine exposure after treatment with LXR ligand GW3965 was unaffected similar to that observed after treatment with ionomycin (Figure 4B). Thereby identifying a role for cyclophilin D in FXR agonist but not in LXR agonist mediated increases in platelet procoagulant activity.

Single platelet analysis of calcium signaling, looking at immobilized individual platelets under arterial shear flow rates (20 dyn/cm^2^ that is equivalent to 1000 s^−1^) identified that when treating platelets with vehicle control, spikes of calcium signaling (intermittent pulses of fluorescence) are observed that are likely to be because of the shear forces the platelets experience under flow.^58^ In contrast, when flowing either GW3965 or GW4064 over the platelets, single platelet calcium signaling was increased and sustained compared with the signaling observed in the presence of vehicle control (Figure 4C and 4D). This increased and sustained signaling is then seen to dissipate over time, which appears to be because of the compromised integrity of the platelet membrane and leakage of the calcium dye from the platelet.

Having identified alterations in calcium signaling after treatment with either LXR or FXR ligands, we set out to determine whether the observed formation of coated platelets was dependent on intracellular or extracellular calcium. Platelets were treated with either GW3965 or GW4064 or vehicle in the presence or absence of BAPTA-AM (10 µmol/L) to chelate intracellular calcium (but leaving extracellular calcium levels unaffected) or EGTA (1 mmol/L), which chelates extracellular calcium. Treatment of platelets with BAPTA-AM prevented the GW3965- and GW4064-induced increases in annexin V binding and fibrinogen binding even after treatment with high concentrations of either ligand, suggesting that the observed phosphatidylserine exposure is dependent on intracellular calcium (Figure 4E). In contrast, treatment of platelets with EGTA did not prevent GW3965- and GW4064-induced increases in fibrinogen binding or P-selectin exposure (annexin V binding not monitored, as annexin V requires presence of Ca^2+^ in the buffer to bind phosphatidylserine), suggesting that LXR and FXR ligand-induced formation of coated platelets is not dependent on extracellular calcium (Figure 4F).

### GW3965 and GW4064 Do Not Activate Apoptotic Pathways

**Figure 5.**
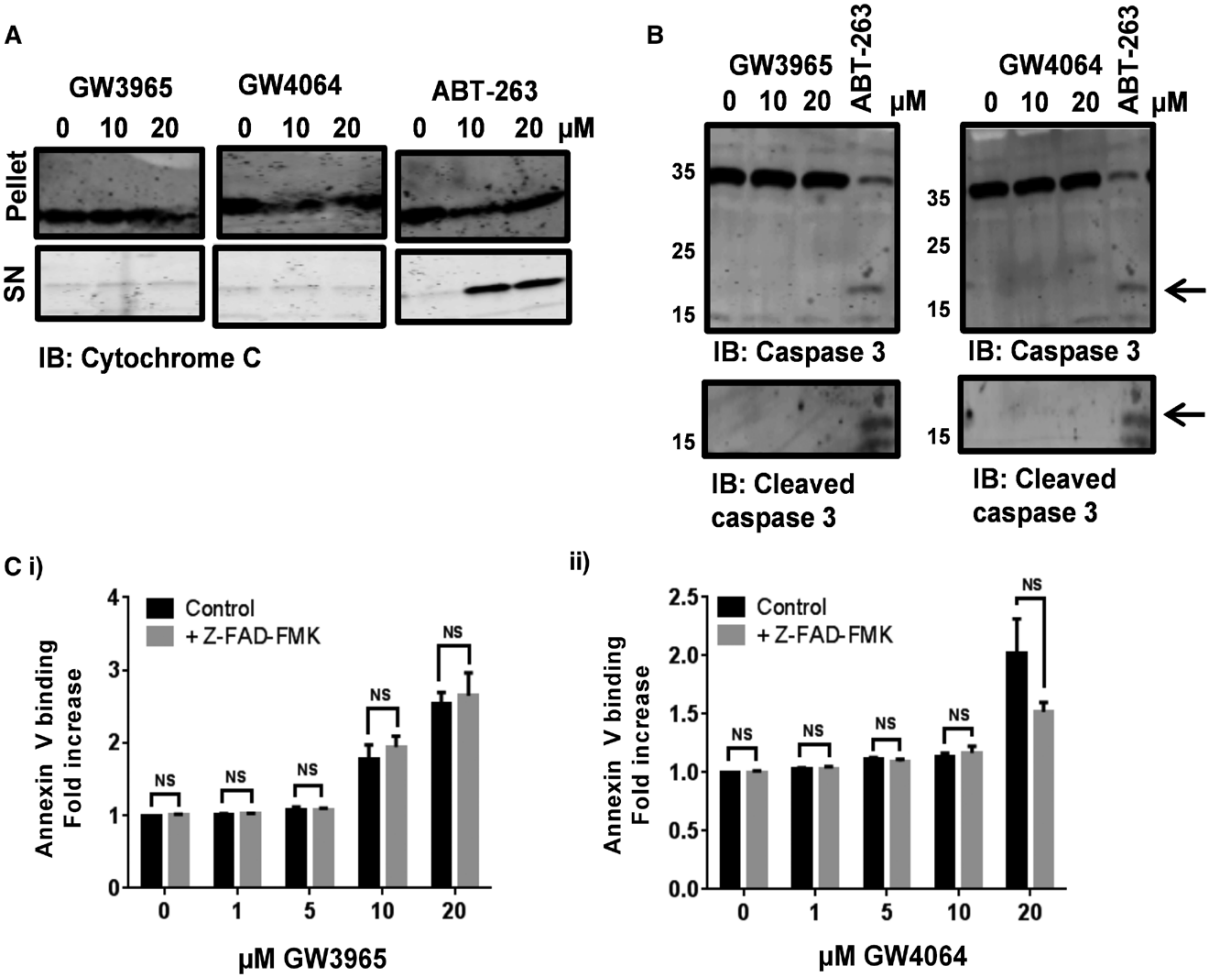
Liver X receptor (LXR) and farnesoid X receptor (FXR) ligands do not initiate apoptosis. **A** and **B**, Human washed platelets were pretreated with GW3965 or GW4064 (10 and 20 mmol/L) or ABT-263 (10 and 20 mmol/L; initiator of apoptosis) for 2 h before (**A**) lysis in mitochondria isolation lysis buffer. The heavy mitochondrial membrane was then isolated from its releasate by centrifugation, and the presence of cytochrome C in the mitochondrial releasate determined by Western blotting. **B**, Activation and cleavage of caspases was also determined in GW3965-treated or GW4064-treated (10 and 20 µmol/L) platelet lysates by Western blotting for the cleaved activated form of the caspases ABT-263 (20 mmol/L) treated platelets were included as a positive control for initation of apoptosis. **C**, Annexin V binding was determined in (i) GW3965-treated and (ii) GW4064-treated platelets after pretreatment with a caspase inhibitor Z-FAD-FMK (10 mmol/L). Representative blots are shown, and results are mean+SEM for n=3 and expressed as fold increase compared with control. *P<0.05 in comparison to vehicle controls.

In other cell types, FXR and LXR agonists have been shown to cause cytotoxicity and initiation of apoptosis.^47–53^ As several of the observed changes induced by GW3965 and GW4064, such as phosphatidylserine exposure and depolarization of the mitochondrial membrane potential, are also observed during cellular apoptosis, the effects of GW3965 and GW4064 on cytochrome c release and caspase activation were determined. As shown in Figure 5, neither was capable of causing the cytosolic release of cytochrome c (a key stage before caspase activation that occurs in apoptosis) or caspase cleavage, in contrast to ABT-263 (10–20 µmol/L), an initiator of apoptosis. This was confirmed by incubation of platelets with a caspase inhibitor Z-FAD-FMK (10 µmol/L) before treatment with GW3965 or GW4064 as Z-FAD-FMK was unable to reverse the potentiation of annexin V binding (Figure 5C), suggesting the observations described here are because of conversion of platelets to the procoagulant state and not a result of initiation of cellular apoptosis.

### LXR and FXR Ligand-Induced Procoagulant Activity Is Dependent on Reactive Oxygen Species

**Figure 6.**
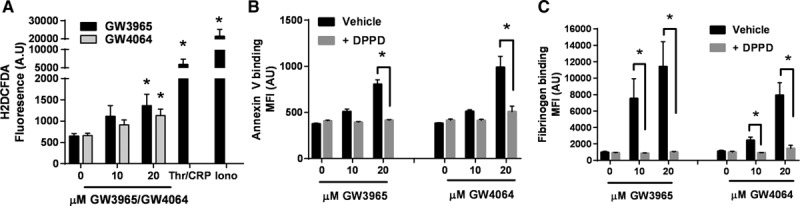
Generation of reactive oxygen species (ROS) is required for induction of procoagulant platelet activity by liver X receptor (LXR) and farnesoid X receptor (FXR) ligands. Human washed platelets were treated for 10 min with or without increasing concentrations of GW3965 (10 and 20 µmol/L) or GW4064 (10 and 20 µmol/L; **A**) in the presence of the fluorescent dye H2DCFDA (an indicator of ROS) and fluorescence measured. Thrombin-treated, CRP-treated, and ionomycin-treated platelets were included as positive controls. **B** and **C**, In the presence or absence of 10 µmol/L DPPD (n,n′-diphenyl-p-phenylenediamine), a reactive oxygen species scavenger before analysis by flow cytometry for (**B**) annexin V binding and (**C**) fibrinogen binding, using an antifibrinogen antibody. Results are mean+SEM for n≥3 and expressed as median fluorescence intensity. *P<0.05 in comparison to vehicle controls.

The generation of reactive oxygen species (ROS) is associated with and dependent on coated platelet formation.^18,19^ To determine whether the mode of action of the LXR and FXR ligands in converting platelets to coated platelets involves ROS generation, the ability of GW3965 and GW4064 to initiate formation of ROS was determined using the fluorescent dye H2DCFDA. Small increases in H2DCFDA fluorescence and ROS generation were observed after treatment with either GW3965 or GW4064 (20 µmol/L; Figure 6A). It has previously been described that antioxidants can prevent Thr/CRP-mediated formation of coated platelets,^18^ and in support of a role for ROS in GW3965- and GW4064-mediated increases in procoagulant coated platelet formation, we found that phosphatidylserine exposure and fibrinogen binding were significantly attenuated in the presence of a ROS scavenger n,n′-diphenyl-p-phenylenediamine (Figure 6B and 6C). These data suggest an important role for ROS in LXR and FXR ligand-dependent coated platelet formation. Given the involvement of ROS but not components of the platelet apoptotic pathways, this suggests that platelets are converted to the procoagulant state via a pathway similar to necrosis, as described previously.^55^

## Discussion

Here, we describe that both LXR and FXR receptor ligands are capable of converting platelets to the procoagulant state at micromolar concentrations that cause inhibition of platelet activity and show receptor specificity in knockout mouse models as previously reported.^25,27^ The effects of the ligands presented here are likely to be physiologically relevant as endogenous LXR and FXR ligands can circulate in the plasma at micromolar concentrations under certain conditions and localized concentrations could be much higher. In a healthy individual, total cholesterol levels (LXR ligands) are maintained <4 mmol/L, but significantly higher levels have been reported in several conditions. Normal levels of bile acids (FXR ligands) in the systemic circulation are reported to be ≥10 µmol/L in posprandial conditions, which can be further increased in disease.^59^

The data presented here are consistent with studies that have found that cholesterol derivatives, LXR receptor ligands, deregulate platelet responses to several platelet agonists^34–36^ and that treatment with bile acids can cause platelet dysfunction,^42^ including membrane vesiculation and platelet swelling.^43,44^ Treatment with either LXR or FXR ligands caused a concentration-dependent increase in phosphatidylserine exposure that was associated with microparticle formation and platelet swelling/ballooning. Increased mean platelet volume is a known marker of platelet hyper-reactivity and associated with increased risk of thrombosis. Increased mean platelet volume has also been observed in several pathological conditions including hyperlipidemia, obesity, high cholesterol, and type 2 diabetes mellitus,^60–63^ conditions associated with increased circulating levels of hydroxycholesterols and bile acids (LXR and FXR agonists). Our findings could, therefore, offer some explanation behind these observations. It is also interesting to note that these observations after treatment with GW3965 and GW4064 also provide a novel example of platelet ballooning and swelling that occurs independently of stimulation by traditional platelet agonists collagen and thrombin.^56^

Concentration-dependent increases in fibrinogen binding (that is not associated with integrin αIIbβ3 activation), α-granule protein exposure at the platelet surface and increased fibrin generation were also observed after treatment with LXR and FXR agonists, indicating the formation of a subset of procoagulant platelets known as coated platelets that are thought to be involved in supporting coagulation reactions. Similar to other previously described examples of increases in procoagulant activity and coated platelet formation, LXR and FXR agonist-dependent increases in procoagulant activity were found to be associated with, and dependent on, sustained intracellular calcium signaling, ROS, and loss of mitochondrial transmembrane potential, all of which have previously been described to be required for Thr/CRP-dependent coated platelet formation.^13,19,20,64,65^ Interestingly, one difference was observed with cyclophilin D, which was found to play a role in FXR agonist-mediated procoagulant platelet formation but was not required for LXR agonist-mediated coated platelet formation.

Despite appearing to be hyper-reactive when in the procoagulant state and able to support the assembly of coagulation complexes that lead to fibrin generation, coated platelets have been shown to undergo closure of integrin αIIbβ3 and other platelet receptors,^20^ reducing the responsiveness of these platelets to platelet stimuli. Coated platelets, therefore, show a significant reduction in their ability to be activated by platelet agonists.^55^ Previous studies have shown that FXR and LXR ligands inhibit platelet aggregation to GPVI agonists and thrombin,^25,27^ and this is further supported by our observations that PAC-1 binding is reduced after treatment with either GW3965 or GW4064 in both CRP- and thrombin-stimulated platelets. It is particularly important to highlight that it is those platelets found to be positive for annexin V binding that show significantly reduced PAC-1 binding compared with vehicle controls, thereby suggesting that procoagulant coated platelet formation is associated with reduced integrin αIIbβ3 activation. Previously published data describe that agonists for both the LXR and FXR receptors attenuate platelet responses to stimulation by both GPVI agonists and thrombin and also inhibit stable thrombus formation after arterial laser injury in vivo.^25,27^ It has been described that during thrombus formation, 2 distinct populations of platelets appear, coaggregated platelets with activated αIIbβ_3_ integrins, which support thrombus growth, and loosely attached platelets that expose phosphatidylserine, which support coagulation but cannot support platelet activation.^20^ We hypothesize that it is this reduction in integrin αIIbβ3 activity observed on coated platelet formation after treatment with either GW3965 or GW4064 that underlies this inhibition of thrombus formation in vivo. We propose that exposure of platelets to LXR and FXR ligands convert platelets to this procoagulant coated state, yet despite their ability to support coagulation, these platelets are desensitized to platelet stimuli through inhibition of integrin αIIbβ3, thereby appearing to negatively regulate platelet functional responses. It is also interesting to note that procoagulant platelets and platelets with increased platelet volume have been shown to be more rapidly cleared from the circulation. This could lead to thrombocytopenia if platelet production is not balanced with platelet turnover,^55,66^ although further study would be required to determine whether this is the case after prolonged exposure to either LXR or FXR ligands.

The work described here has significant clinical implications as circulating and localized concentrations of several agonists for these nuclear receptors, including cholesterol derivatives and bile acids, are increased under several pathophysiological conditions. These nongenomic effects of nuclear receptor ligands could, therefore, contribute to platelet deregulation. Ligands for LXR and FXR are under clinical development for the treatment of diabetes mellitus, high cholesterol, and obesity; and it is therefore important to balance their beneficial metabolic effects against their possible side effects on platelet reactivity and function.

## Acknowledgments

We would like to thank Dr C. Dubois, Aix Marseille University, France, and Dr L.M. Holbrook, University of Reading, United Kingdom, for the provision of reagents. A.J. Unsworth designed the research, performed experiments, analyzed results, and wrote the article. A.P. Bye, D.S. Tannetta, M.J.R. Desborough, N. Kriek, T. Sage, H.E. Allan, and M. Crescente performed experiments and analyzed results. P. Yaqoob and T.D. Warner designed experiments. C.I. Jones and J.M. Gibbins designed the research and wrote the article.

## Sources of Funding

This work was supported by the British Heart Foundation (RG/09/011/28094, RG/15/2/31224, PG/16/36/31967, and PG/14/48/30916); the Wellcome Trust (101604/Z/13/Z); a Queen Mary University of London college PhD studentship; and the Medical Research Council (MR/J002666/1).

## Disclosures

None.

## Supplementary Material

## Notes

Highlights

- Treatment of platelets with liver X receptor or farnesoid X receptor ligands converts platelets to the procoagulant state and forms coated platelets.
- Liver X receptor and farnesoid X receptor ligand-induced coated platelets display increased phosphatidylserine exposure, platelet swelling, microparticle formation, increased P-selectin exposure, fibrinogen binding, and fibrin generation that is supported by increased serine protease activity.
- These coated platelets are, however, desensitized to platelet stimuli through receptor closure and cleavage, which is consistent with their ability to inhibit platelet function and stable thrombus formation in vivo.
