# Supplementary material for: Farnesoid X Receptor and Liver X Receptor Ligands Initiate Formation of Coated Platelets
Source: Arterioscler Thromb Vasc Biol. 2017 Jul 26;37(8):1482–93. doi: 10.1161/ATVBAHA.117.309135 (PMC5526435; doi:10.1161/ATVBAHA.117.309135)
Supplement: Supplementary file 1 [file atv-37-1482-s001.pdf]

FXR and LXR ligand dependent formation of COATED platelets

Supplemental figures.

Supplementary Figure I. LXR and FXR agonists initiate phosphatidylserine exposure and microparticle formation

Human washed platelets were treated for 10 minutes with or without GW3965 (20 µM) or GW4064 (20 µM) or vehicle control before analysis by flow cytometry for A) Annexin V binding and B and C) Microparticle formation. A) Gating strategy for selecting Annexin V positive cells. B) Microparticle gating strategy shown. Microparticles were determined by gating for the microparticle population using forward and side scatter profiles using a Canto II flow cytometer set up. i) FSC vs. SSC plot of ApogeeMix beads and position of microparticle (MP) gate, set using 500nm latex beads. ii) FITC vs SSC plot confirming the identification of the FITC fluorescent 500nm latex beads. C) Representative i) FSC vs. SSC plots and ii) APC-A fluorescence of PS exposure (Annexin V bound) histograms of platelet derived microparticles following treatment with ionomycin. D) Representative APC-A fluorescence of PS exposure (Annexin V bound) histograms of platelet derived microparticles following treatment with i) GW3965 or ii) GW4064.

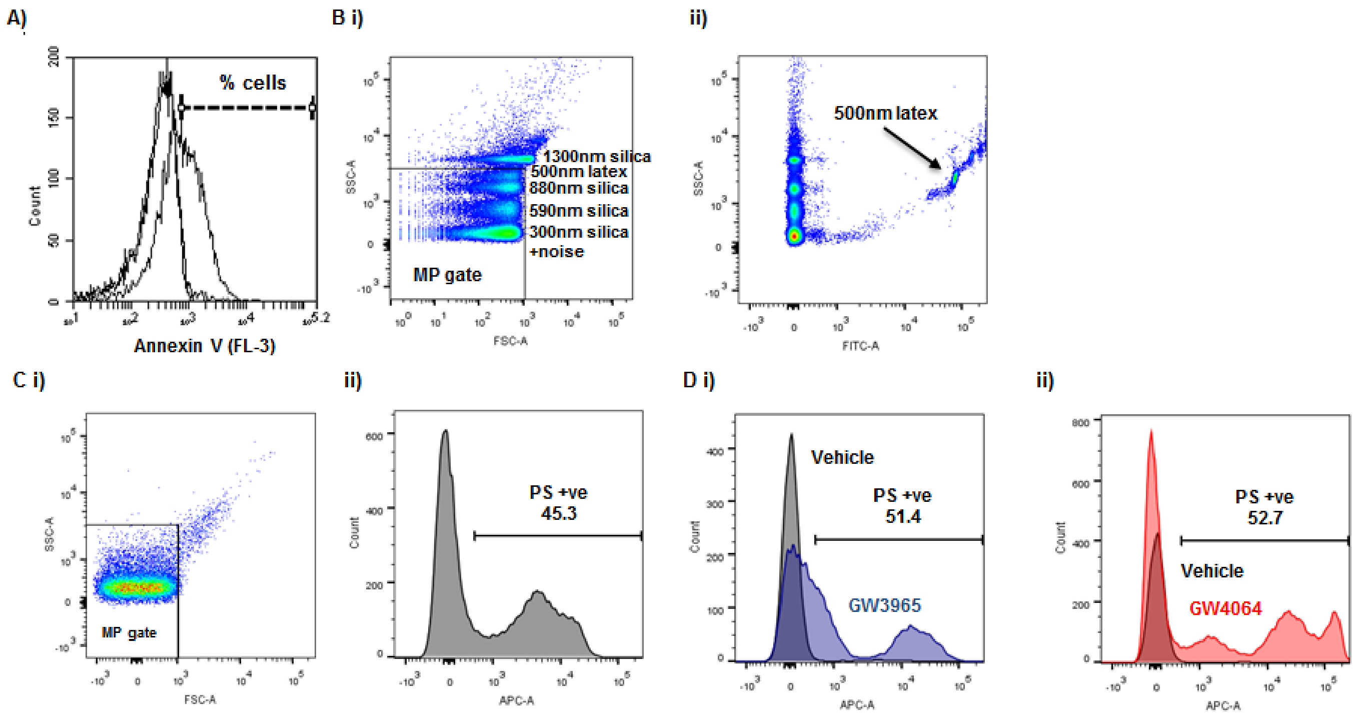

Supplementary Figure II. LXR and FXR agonists initiate platelet swelling.

Human washed platelets were treated for 10 minutes with or without i) GW3965 (40 µM), ii) GW4064 (40 µM), iii) Thrombin (0.1 U/mL), iv) Ionomycin (5 µM) or vehicle control in the presence of absence of integrillin (10 µM) before platelet swelling measured by analysing light transmission was determined. An increase in light transmission is associated with increased platelet swelling. A) Representative images of light transmission over time, black lines represent in the absence of integrillin, dotted lines represent in the presence of integrillin. B) data expressed as % of light transmission. C) platelet count. Results are mean + S.E.M. for n≥3, * indicates p<0.05 in comparison to vehicle controls unless stated otherwise.

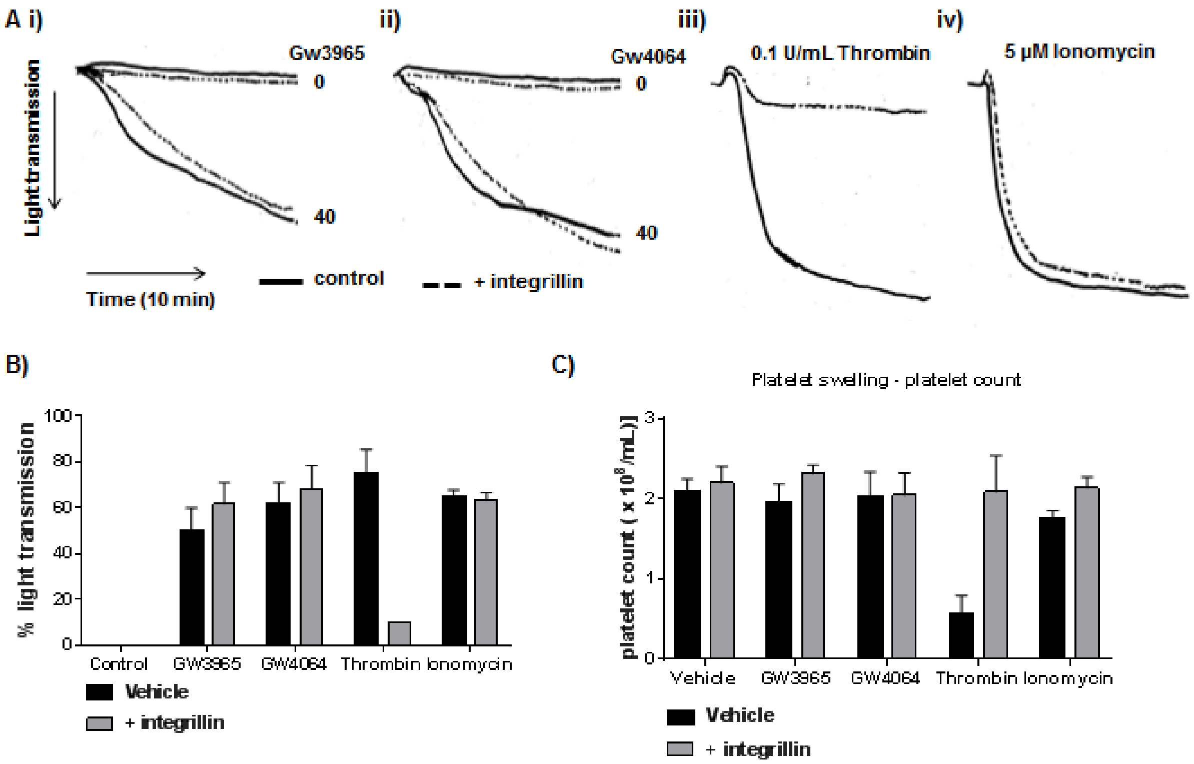

Supplementary Figure III. LXR and FXR agonists increase Annexin V and fibrinogen binding.

Human washed platelets were treated for 10 minutes with or without increasing concentrations of A) 27-hydroxycholesterol or 24S-hydroxycholesterol (10, 30, 100 µM) or B) 6-Ethylchenodeoxycholic acid (6-ECDCA) or chenoxydecholic acid (CDCA) (100, 300 µM) or vehicle control before analysis by flow cytometry for i) Annexin V binding which is a measure of phosphatidylserine exposure or ii) fibrinogen binding, data expressed as percentage of annexin V or fibrinogen positive events. Results are mean + S.E.M. for n≥3, * indicates $p<0.05$ in comparison to vehicle controls unless stated otherwise.

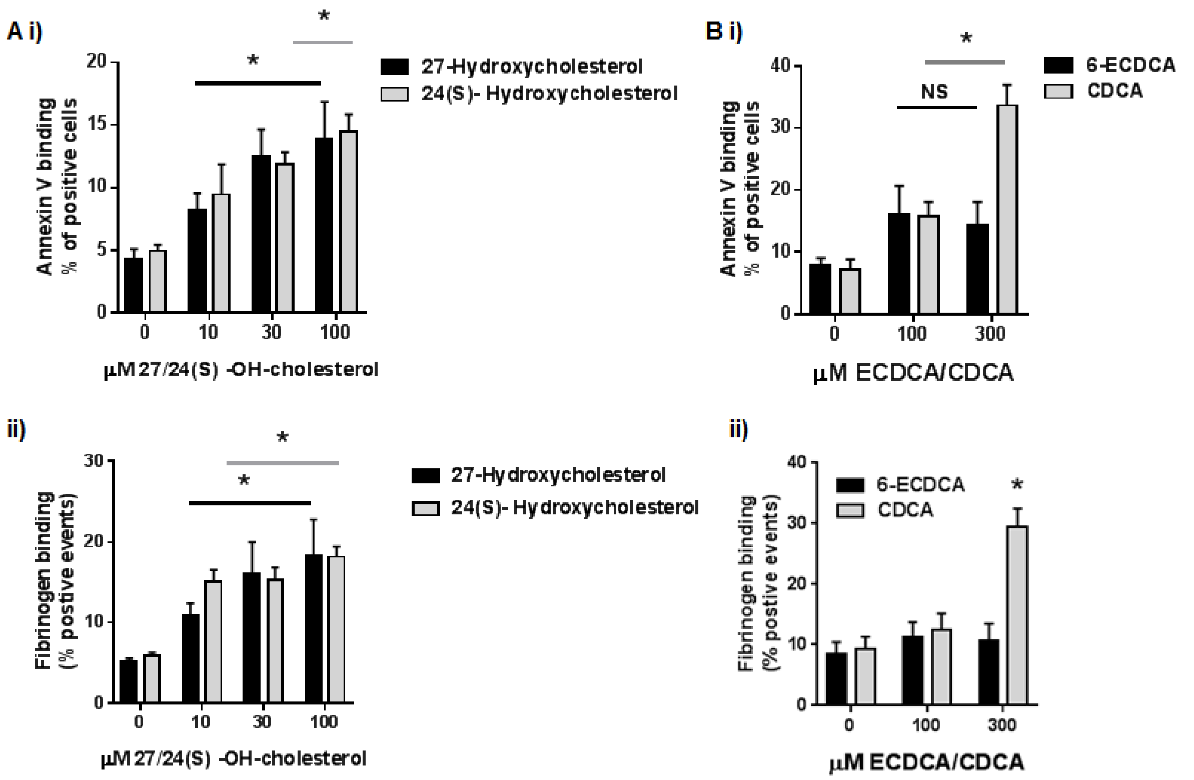

Supplemental Figure 4. GW3965 and GW4064 increase platelet Annexin V and fibrinogen binding.

Human washed platelets ($8x10^7$ cells/ml) were incubated for 20 minutes with fluorescein isothiocyanate-labelled (FITC) anti-fibrinogen (blue), Alexa-647 conjugated anti-Annexin V (red) and PE Cy conjugated anti-CD42b antibodies (green) in the presence of A) vehicle control (DMSO), B) GW3965 (20 µM) or C) GW4064 (20 µM) and platelets visualised using an ImageStream^x MarkII imaging flow cytometer. 3 representative images from 3 separate experiments shown.

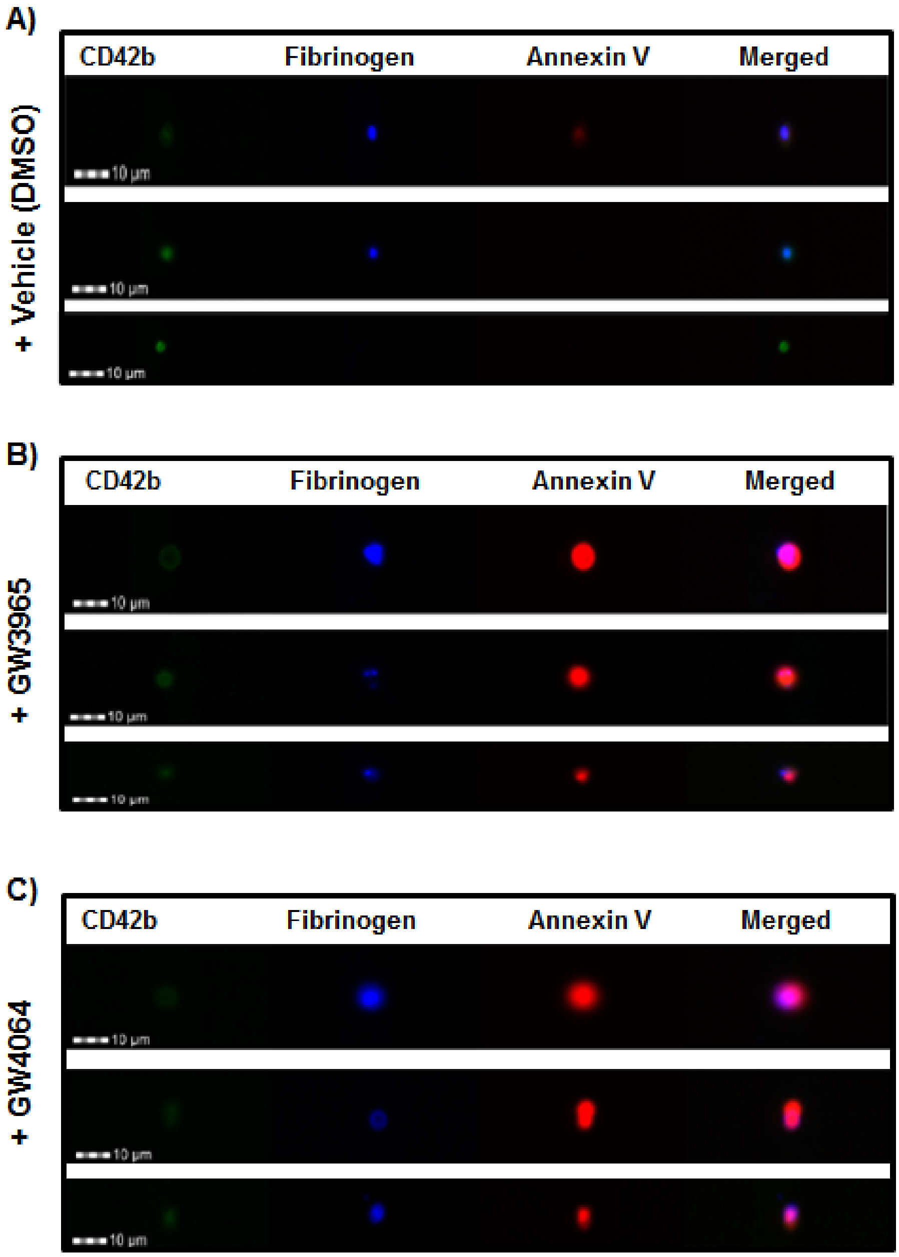

Supplemental Figure V. GW3965 and GW4064 speed up the kinetics of thrombin generation in platelet rich plasma.

Calibrated automated thrombography was performed on platelet rich plasma using a tissue factor trigger (final concentration 1 pM) following treatment with, vehicle (0.1 % DMSO), GW3965 (100 µM) or GW4064 (100 µM) (higher concentrations used to adjust for plasma binding) for 20 minutes before analysis. A) representative traces of thrombin generation over time shown following treatment with vehicle or i) GW3965 or ii) GW4064. B) Time to peak thrombin generation (time from coagulation activation to peak thrombin generation).Results where relevant represent mean + S.E.M. for n=5, * indicates p≤0.05 in comparison to vehicle controls.

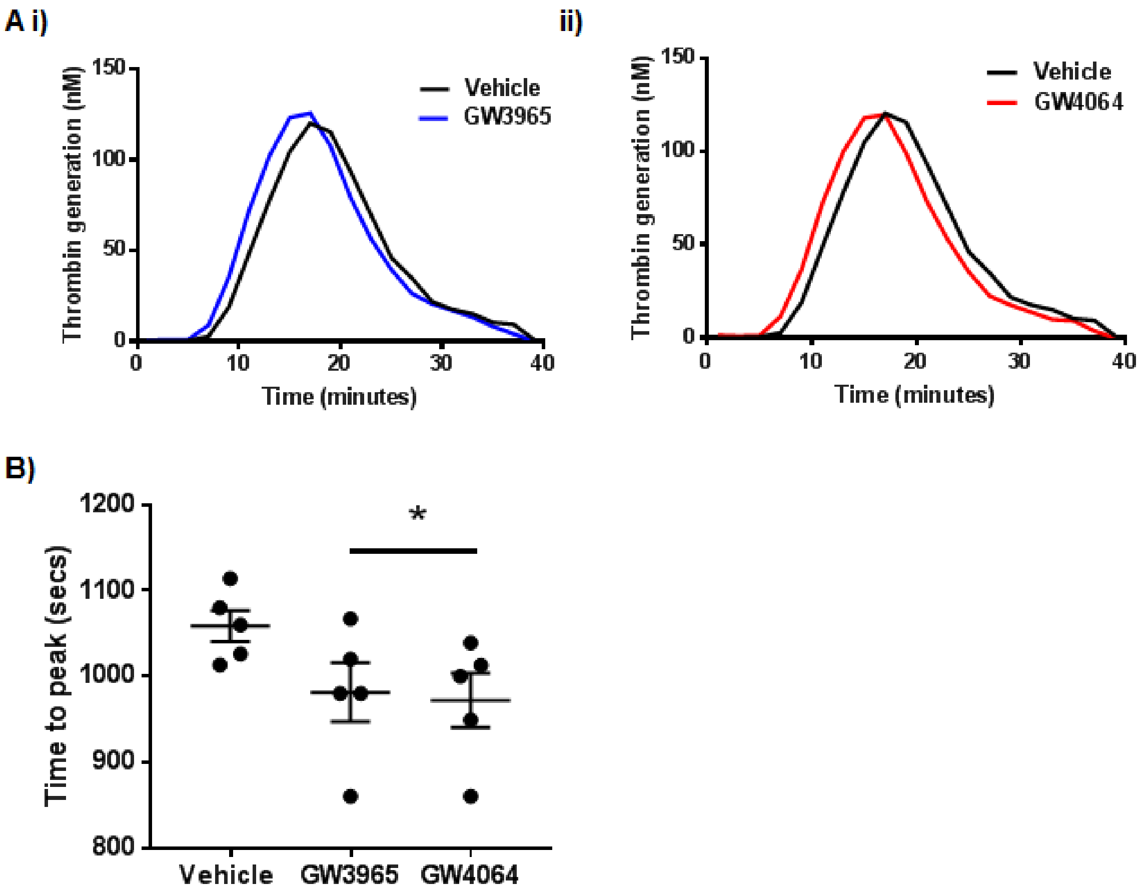

Supplemental Figure VI. GW3965 and GW4064 inhibit platelet adhesion on fibrinogen.

Human washed platelets pretreated for 10 minutes with or without GW3965 (10, 20 µM), GW4064 (10, 20 µM) or vehicle control were A) stimulated with i) 1 µg/mL CRP or ii) Thrombin (0.1 U/mL) and then analysed for integrin αIIbβ3 activation by monitoring PAC-1 binding using flow cytometry. Data expressed as median fluorescent intensity. B. C, D) exposed to fibrinogen (100 µg/mL) coated coverslips. B,C) left to adhere and spread on fibrinogen (100 µg/mL) coated coverslips. i) Representative images of spreading and adhesion after 45 min in vehicle and treated samples. Platelets were stained with phalloidin Alexa-488 for visualisation. Images were taken under oil immersion with magnification ×100. ii) Adhesion: number of platelets adhered were counted in 5 randomly selected fields of view and the number of cells adhered expressed as a percentage of the vehicle treated control. iii) Spreading: platelets were classified into 3 different categories to determine the extent of their spreading (Adhered but not spread, Filopodia: platelets in the process of extending filopodia and Lamellipodia: platelets in the process of extending lamellipodia including those fully spread) at least 100 platelets of each type were scored. Results expressed as relative frequency, as a percentage of the total number of platelets adhered. D) Analysis of Annexin V binding by flow cytometry of non-adhered platelets following exposure to to fibrinogen (100 µg/mL) coated coverslips for 45 minutes. E) analysed for talin cleavage (a marker of integrin activation, in platelet lysates was determined by western blotting), ionomycin (5 µM) was included as a positive control. Representative images shown. Results where relevant represent mean + S.E.M. for $n \geq 3$, * indicates $p \leq 0.05$ in comparison to vehicle controls.

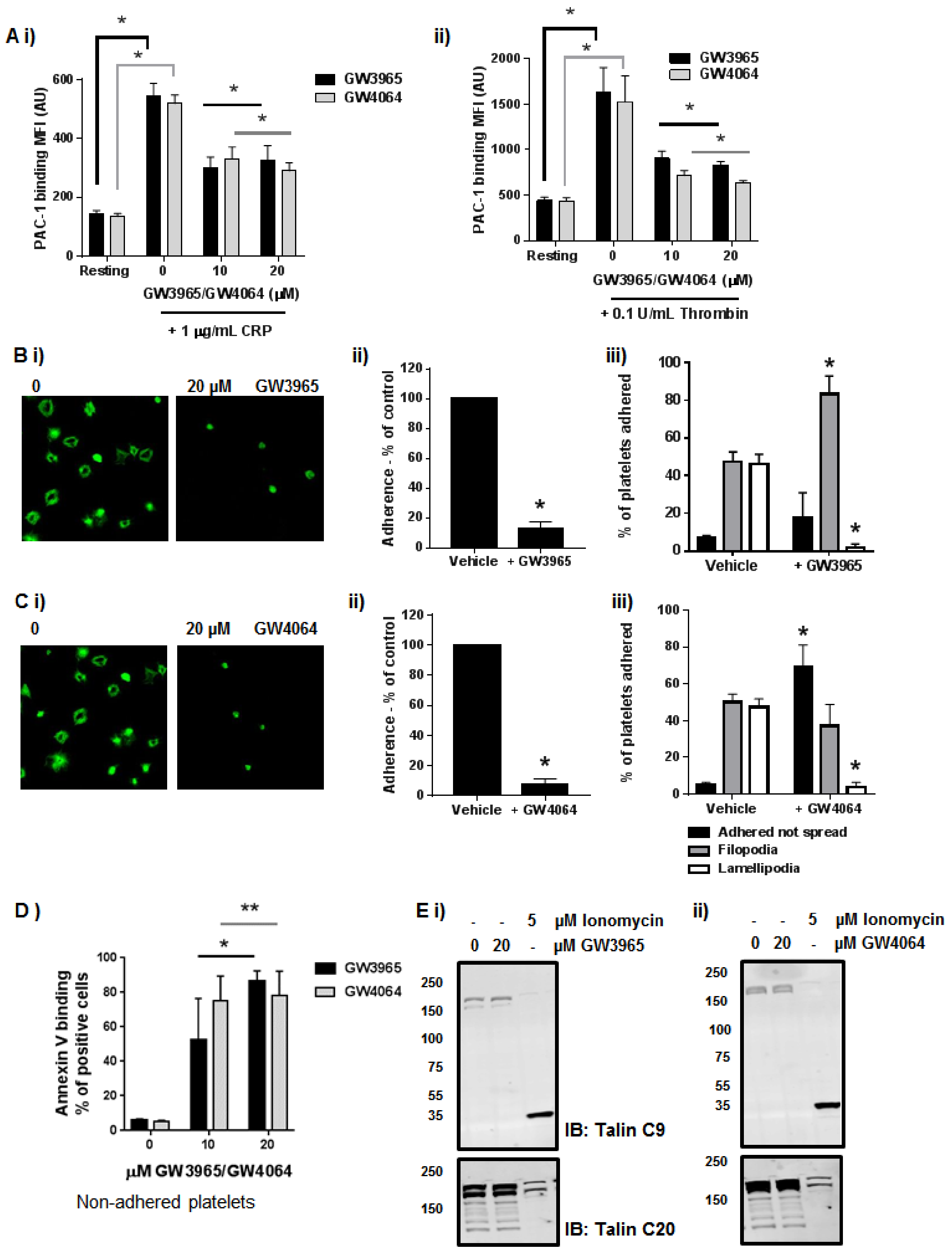

A i)
PAC-1 binding MFI (AU)
GW3965
GW4064
Resting
GW3965/GW4064 (µM)
+ 1 µg/mL CRP
ii)
PAC-1 binding MFI (AU)
+ 0.1 U/mL Thrombin
B i)
20 µM GW3965
Adherence - % of control
Vehicle
+ GW3965
iii)
% of platelets adhered
C i)
20 µM GW4064
+ GW4064
Adhered not spread
Filopodia
Lamellipodia
D)
Annexin V binding % of positive cells
µM GW3965/GW4064
Non-adhered platelets
E i)
µM Ionomycin
µM GW3965
IB: Talin C9
IB: Talin C20
µM GW4064
