# Supplementary material for: Farnesoid X Receptor and Liver X Receptor Ligands Initiate Formation of Coated Platelets
Source: Arterioscler Thromb Vasc Biol. 2017 Jul 26;37(8):1482–93. doi: 10.1161/ATVBAHA.117.309135 (PMC5526435; doi:10.1161/ATVBAHA.117.309135)
Supplement: Supplementary file 2 [file atv-37-1482-s002.pdf]

**FXR and LXR ligand dependent formation of COATED platelets.**

## MATERIALS AND METHODS

### Reagents

Nuclear Receptor ligands, GW3965 and GW4064 were purchased from Sigma Aldrich (Poole, UK). BAPTA, DPPD (N-N Diphenyl-p-phenylenediamine), Z-Gly-Gly-Arg-AMC fluorescent substrate and thrombin were purchased from Sigma Aldrich (Poole, UK), ABT-263 from Selleck Chem (Newmarket, UK) and Z-VAD-FMK from BD Biosciences (Oxford, UK). CRP-XL from Prof. R Farndale (University of Cambridge, UK). Fluorescein isothiocyanate-labelled (FITC) anti-fibrinogen antibody was purchased from Dako, PE/Cy5 anti-human CD62P (P-selectin), PE conjugated anti-CD42b antibody and Cy5.5 labelled Annexin V were purchased from BD Biosciences (Oxford, UK). Anti- caspase 3 and cleaved caspase 3 antibodies were purchased from New England BioLabs (Cell Signalling) (Hitchin, UK), 14-3-3 and actin (C20) antibodies were purchased from Santa Cruz Biotechnology, USA), anti-fibrin antibody was a gift from C. Dubois and conjugated to Alexa-647 in house. Fluorophore conjugated secondary antibodies, Alexa-647 labelled Annexin V and Fluo-4 calcium indicator dye were purchased from Life Technologies (Paisley, UK).

### Human washed platelet preparation

Human blood was taken from consenting aspirin free healthy volunteers. Blood was collected into a syringe containing 4% (weight/volume) sodium citrate and acid citrate dextrose (ACD) was added prior to preparation of platelet rich plasma (PRP). Washed platelets were prepared by first isolating the PRP following centrifugation at 100 xg for 20 minutes at room temperature, and centrifugation twice at 1000 xg for 10 minutes to pellet the platelets in the presence of 1.25μg/mL prostacyclin ($PGI_2$) as described previously [1]. Platelets were resuspended in modified Tyrode's-HEPES buffer, (134mM NaCl, 0.34mM $Na_2HPO_4$, 2.9mM KCl, 12mM $NaHCO_3$, 20mM N-2-hydroxyethylpiperazine-N′-2-ethanesulfonic acid, 5mM glucose and 1mM $MgCl_2$, pH 7.3) and rested for 30 minutes at 30°C before experiments.

### Measuring fibrinogen binding, alpha-granule secretion, fibrin binding, membrane integrity and phosphatidylserine exposure by flow cytometry

Flow cytometry was used to examine affinity up regulation of the integrin αIIbβ3, alpha granule secretion, fibrin binding, membrane integrity and phosphatidylserine exposure by detecting levels of fibrinogen binding to integrin αIIbβ3, P-selectin exposure on the platelet surface, fibrin bound to the platelet surface, calcein permeability and Annexin V binding respectively in human washed platelets. Following the addition of the nuclear receptor agonists, platelets were incubated at room temperature for 20 minutes with either fluorescein isothiocyanate-labelled (FITC) anti-fibrinogen antibody, PE/Cy5 anti-human CD62P (P-selectin), Alexa-647 conjugated anti-fibrin antibody and Cy5.5 labelled Annexin V or pre loaded with calcein-AM. Antibodies were all used at saturating concentrations, and the instrument settings were kept the same throughout the experiments. Reactions were stopped by a 1 in 5 dilution in 0.2% (v/v) paraformaldehyde or HEPEs buffered saline. Flow cytometric acquisition was performed using a BD Accuri C6 flow cytometer, and data were collected from 10,000 events in the platelet gate (determined by Forward and side scatter profiles) and analysed by calculating median fluorescence intensity and percentage of positive cells using CFlow Sampler software.

### Flow cytometric analysis of platelet derived microparticles

Analysis of the generation of microparticles (MP) following treatment of washed platelets (2 x $10^7$ /mL) with GW3965, GW4064 or Ionomycin was carried out by flow cytometry, using a BD Canto II Flow Cytometer (BD Biosciences, Oxford UK) set to trigger on side scatter (SSC) and all data were analysed using FlowJo software. ApogeeMix beads (Apogee Flow Systems, Hemel Hempstead UK), comprised of 180nm, 240nm, 300nm, 590nm, 880nm and 1300nm silica beads and 110nm and 500nm green fluorescent latex beads, were used to determine the optimum SSC and forward scatter (FSC) voltages for microparticle detection. The microparticle gate was set using the 500nm latex beads (Supplemental Fig 1B) [2]. To acquire data, events were collected over 2 mins and a BD Trucount tube was run at the end of each session to measure the volume of sample analysed. Prior to running samples, Modified-Tyrodes- HEPEs buffer was also run for 2 mins to assess the level of background events. Prior to use, annexin V conjugated to APC (Ann V-APC; Life Technologies) was filtered using Millipore 0.1µm centrifugal devices (Merck Millipore, Feltham UK) to minimalize interference by background microparticles. Ann V-APC was also titrated to ensure use at the optimum concentration. Modified-Tyrodes- HEPEs buffer and annexin V-APC (4 µL) alone controls were run to measure background contaminating events. Unlabeled samples were also run to set the gate for annexin V-APC positive microparticles to include ≤1% of background events. Samples were then incubated with annexin V-APC (1:200 dilution) for 15min at R/T in the dark and then acquired for 2 min on the flow cytometer. The number of annexin V positive events in the GW3965 and GW4064 treated sample was then counted following gating using a vehicle treated annexin v-APC labelled control for comparison.

**Platelet swelling assay**

Washed platelets were prepared from human blood and platelet swelling measured by optical transmission in an aggregometer (Chronolog Corp., Havertown, PA, USA) as described previously [2]. An increase in light transmission without aggregate formation and alteration in platelet count is indicative of platelet swelling. Platelet counts were monitored after treatment to confirm lack of cell lysis or aggregation [2]. Mean platelet volume was determined by using an ACT5 haematology analyser (Beckman Coulter Inc, UK).

**Measuring mitochondrial membrane potential by flow cytometry.**

The integrity of platelet mitochondrial membranes was measured in washed platelets preloaded with the fluorescent dye JC-1 and the FL1/FL2 ratio for each sample measured by flow cytometry. Samples were prepared as described in the earlier flow cytometry section. Flow cytometric acquisition was performed using a BD Accuri C6 flow cytometer, and data were collected from 10,000 events in the platelet gate (determined by Forward and side scatter profiles) and analysed using CFlow Sampler software. An increase in the ratio compared to control indicates hyperpolarisation of the mitochondrial membrane whilst a decrease in the FL2/FL1 ratio indicates membrane depolarisation [3].

**Single platelet $Ca^{2+}$ imaging.**

PRP was loaded with Fluo-4 AM (2 µM) for 1h at 30°C and then washed by centrifugation at 350 *xg* for 20 mins and resuspended in Tyrode's-HEPEs buffer in the presence of 1mM $CaCl_2$. Platelets were then attached to mouse anti-human PECAM-1 antibody (WM59) coated glass-bottom Vena8 GCS biochips (Cellix Ltd., Dublin, Ireland), which had been pre-incubated with 2% BSA for 1 hour at 37°C to prevent glass-induced platelet activation. Vehicle, GW3965 (20µM) or GW4064 (20 µM) were then flowed through the chips at low shear rate (400 $s^{-1}$) and calcium signalling monitored by observing fluorescence measurements with excitation at 488 nm and emission at 525 nm with a 60x magnification lens using a Nikon A1-R confocal microscope.

**Immunofluorescence microscopy.**

Human platelets treated with or without GW3965 (20µM) or GW4064 (20 µM) and fixed in 0.2% formalin were left to settle on poly-L-lysine coverslips for 1 hour at 37ºC before blocking (1% BSA, 2% donkey serum). Coverslips were then incubated with an Alexa 488nm fluorophore conjugated anti-fibrinogen antibody and Cy5.5 labelled Annexin V for 1 hour at room temperature in the dark. Coverslips were washed and mounted onto slides. Platelets were imaged with a 100 x magnification oil immersion lens on a Nikon A1-R confocal microscope. Data analysed using ImageJ software.

**Imaging flow cytometry**

For additional imaging of coated platelet formation, an ImageStream$^X$ Mark II imaging flow cytometer (Amnis Corporation, WA, USA) was used because this combines flow cytometry with high content image analysis. To prepare samples, washed human platelets ($8x10^7$ cells/ml) were pre-treated with vehicle (0.1% DMSO), GW3965 (20 µM) or GW4064 (20 µM) for 20 minutes and stained with anti-CD42b-PE to enable detection of the platelet population, anti-fibrinogen- FITC and annexin-V conjugated to Alexa-647 and then diluted 10-fold in modified Tyrodes buffer. The ImageStream$^X$ Mark II imaging flow cytometer was then used to acquiring up to 12 images of cell including, brightfield, scatter, and multiple fluorescent images. Briefly, the platelet population was gated by size and PE- CD42b positivity and confirmed visually by acquired images. Data for 10 000 events were collected and analysed using IDEAS software (Amnis, Seattle, WA)

**Serine protease activity assay**

Human washed platelets were preincubated with Z-Gly-Gly-Arg-AMC fluorescent substrate prior to addition of nuclear receptor agonists, using a 96 well plate assay approach and fluorescence plate reader. Increases in levels of fluorescence as a result of serine protease activity and cleavage of the AMC group was monitored at 37°C for 1 hour at an excitation wavelength of 366nm and emission wavelength of 460nm.

**Thrombin generation assay**

Calibrated automated thrombography was performed on platelet rich plasma using a tissue factor trigger (final concentration 1 pM). Vehicle (0.1% DMSO), GW3965 (100 µM) or GW4064 (100 µM) was added to platelet rich plasma 20 minutes before analysis. Coagulation was initiated with calcium chloride and was continuously registered using a fluorogenic substrate (ZGGR-AMC; FluCa reagent). Thrombin generation was measured against a thrombin calibrator using an automated fluorometer (Fluoroskan Ascent®, ThermoLabsystem, Helsinki, Finland) and output was analysed using Thrombinoscope software (Thrombinoscope BV).

**Immunoblotting**

SDS-PAGE and immunoblotting were performed using standard techniques as described previously [1]. Proteins were detected using antibodies raised against the protein of interest and fluorophore-conjugated secondary antibodies and then visualised using a Typhoon imaging system (GE Healthcare).

**Statistical analysis**

Statistical analyses were carried out on data using GraphPad prism software. When comparing two sets of data, an unpaired, 2-tailed Student's t test was used. If more than two means were present, significance was determined by one way ANOVA. Where data is normalised, statistical analysis was performed prior to normalisation and also using the non-

parametric Wilcoxon signed-rank test. P≤0.05 was considered statistically significant. Unless stated otherwise, values are expressed as mean ±SEM.


1. Kaiser WJ, Holbrook LM, Tucker KL, Stanley RG, and Gibbins JM, A functional proteomic method for the enrichment of peripheral membrane proteins reveals the collagen binding protein Hsp47 is exposed on the surface of activated human platelets. J Proteome Res, 2009; 8(6):2903-14.
2. Mattheij NJ, Gilio K, van Kruchten R, et al., Dual mechanism of integrin alphaIIbbeta3 closure in procoagulant platelets. J Biol Chem, 2013; 288(19):13325-36.
3. Verhoeven AJ, Verhaar R, Gouwerok EG, and de Korte D, The mitochondrial membrane potential in human platelets: a sensitive parameter for platelet quality. Transfusion, 2005; 45(1):82-9.
