# Supplementary figures and images for: Farnesoid X Receptor and Liver X Receptor Ligands Initiate Formation of Coated Platelets
Source: Arterioscler Thromb Vasc Biol. 2017 Jul 26;37(8):1482–93. doi: 10.1161/ATVBAHA.117.309135 (PMC5526435; doi:10.1161/ATVBAHA.117.309135)

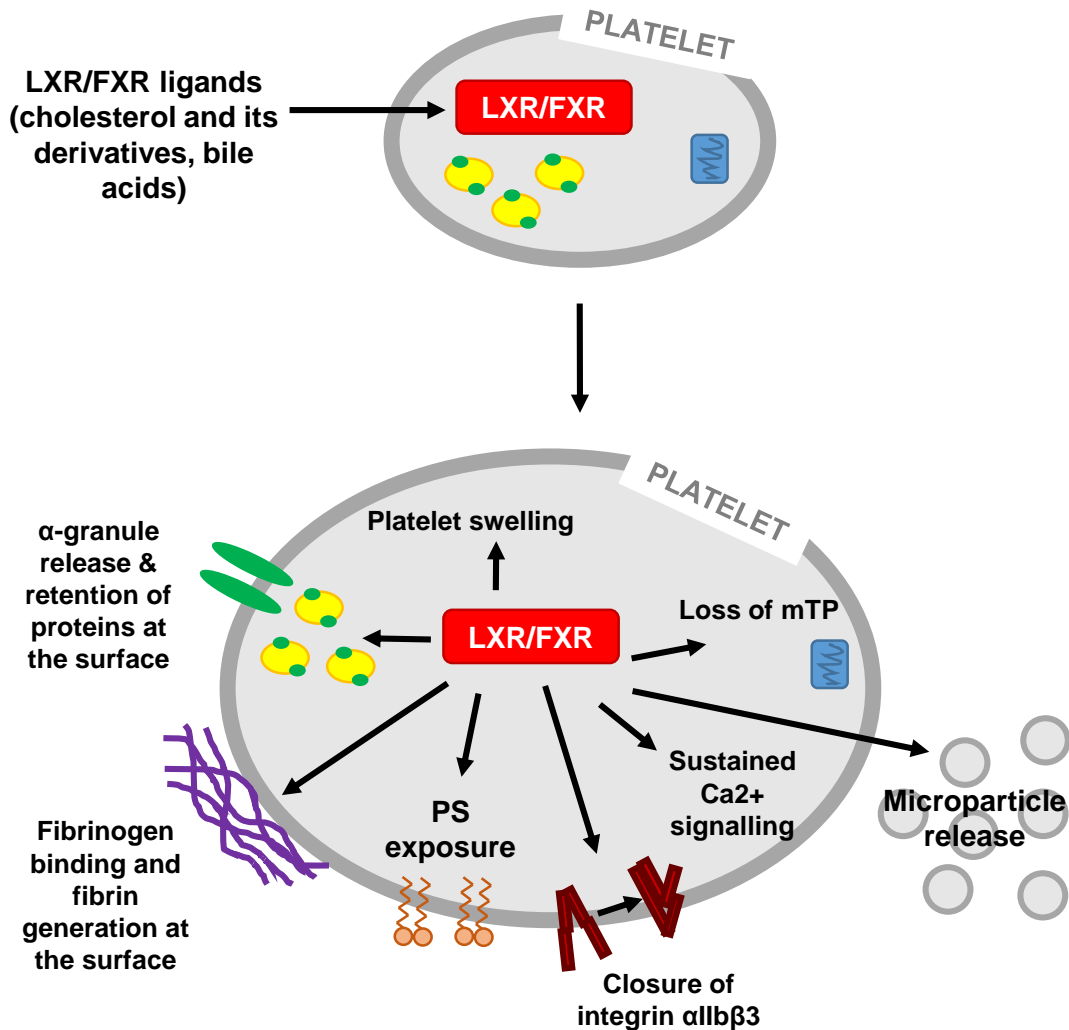

Supplement: Supplementary file 3 [file atv-37-1482-s003.pdf]
